# Identification of Potential Inhibitors of Calcium/Calmodulin-Dependent Protein Kinase IV from Bioactive Phytoconstituents

**DOI:** 10.1155/2020/2094635

**Published:** 2020-07-16

**Authors:** Preeti Gupta, Shama Khan, Zeynab Fakhar, Afzal Hussain, Md. Tabish Rehman, Mohamed F. AlAjmi, Asimul Islam, Faizan Ahmad, Md. Imtaiyaz Hassan

**Affiliations:** ^1^Centre for Interdisciplinary Research in Basic Sciences, Jamia Millia Islamia, Jamia Nagar, New Delhi 110025, India; ^2^Department of Clinical Microbiology and Infectious Diseases, School of Pathology, University of the Witwatersrand, Johannesburg 2193, South Africa; ^3^Molecular Sciences Institute, School of Chemistry, University of the Witwatersrand, WITS, 2050 Johannesburg, South Africa; ^4^Department of Pharmacognosy, College of Pharmacy, King Saud University, Riyadh 11451, Saudi Arabia

## Abstract

Calcium/calmodulin-dependent protein kinase IV (CaMKIV) is an upstream regulator of CaMKK-CaMKIV signaling cascade that activates various transcription factors, thereby regulating several cellular activities including, neuronal communication and immune response. Owing to the abnormal expression in cancer and neurodegenerative diseases, the CaMKIV has been considered a potential drug target. In the present study, we checked the binding affinity of plant-derived natural compounds viz., quercetin, ellagic acid (EA), simvastatin, capsaicin, ursolic acid, DL-*α*-tocopherol acetate, and limonin towards CaMKIV. Molecular docking and fluorescence binding studies showed that EA and quercetin bind to the CaMKIV with a considerable affinity in comparison to other compounds. Enzyme inhibition assay revealed that both EA and quercetin inhibit CaMKIV activity with their IC_50_ values in the micromolar range. To get atomistic insights into the mode of interactions, inhibition mechanism, and the stability of the CaMKIV-ligand complex, a 100 ns MD simulation analysis was performed. Both EA and quercetin bind to the catalytically important residues of active site pocket of CaMKIV forming enough stabilizing interactions presumably inhibiting enzyme activity. Moreover, no significant structural change in the CaMKIV was observed upon binding of EA and quercetin. In conclusion, this study illustrates the application of phytoconstituents in the development of therapeutic molecules targeting CaMKIV having implications in cancer and neurodegenerative diseases after *in vivo* validation.

## 1. Introduction

Calcium/calmodulin-dependent kinase IV (CaMKIV), a member of the Ser/Thr protein kinase family, is an integral part of Ca^2+^-triggered CaMKK-CaMKIV signaling cascade where it regulates the phosphorylated-mediated activation of various transcription activators, such as JUN, CREB1, MEF2D, and RORA [1]. These downstream targets of CaMKIV further regulate various physiological processes including neuronal communication, inflammation, immune response, and memory consolidation [2]. It regulates cell motility, survival, cell cycle progression, and apoptosis via phosphorylation/dephosphorylation events [3, 4]. CaMKIV shows high expression levels in the cerebellum, thymus, T-lymphocytes, and testis [5, 6]. Under the basal conditions, the activation of CaMKIV is in the fine regulation of intracellular Ca^2+^ concentration. Increased intracellular free calcium stimulates a signaling cascade whereby a ubiquitously expressed protein calmodulin (CaM) binds to Ca^2+^, and Ca^2+^/CaM complex further interacts with CaMKIV and alters its functionality [7]. Intracellular Ca^2+^ levels have been identified as a crucial factor in the regulation of wide arrays of cell death modalities such as necrosis, necroptosis, and apoptosis [8, 9]. It has been established that oxidative stress promotes the activation of CaMKIV which in turn induces the activation of antiapoptotic signaling cascades including ERK, AKT, and NF-*κ*B in various cell types. In conjunction with this, Rodriguez-Mora et al. has found that the inhibition of CaMKIV enhances the H_2_O_2_-induced apoptosis in breast cancer (MCF-7) cells [10].

Many reports have shown the abnormal expression of CaMKIV in various malignancies, including cancer of the lung, prostate, liver, and ovary [11–13]. In addition, the overexpression of CaMKIV is associated with systemic lupus erythematosus, cerebral hypoxia, ischemic stroke, and infectious diseases [14–16]. The involvement of CaMKIV and related kinases in the progression of cancers and neurological diseases makes it an attractive drug target [1, 17].

To date, chemotherapy is a common mode for cancer therapy. However, a concoction of drugs used in chemotherapy imposes serious threats upon long-term usage. Thus, it is imperative to devise alternative strategies that are effective in handling cancer and at the same time are clinically safe. For decades, plant-derived natural products have been investigated as promising therapeutic agents to combat cancer, neurodegenerative and cardiovascular diseases [18–20]. Natural compounds evoke diverse processes such as autophagic cell death, apoptosis, and programmed necrosis to elicit anticancerous activity [19, 21]. The active ingredients of plant-derived formations are known to possess antioxidant, antidiabetic, anti-inflammatory, antibacterial, antiviral, and hepatoprotective effects [22–24]. Moreover, natural products overcome the disadvantages associated with conventional chemotherapy such as limited bioavailability and undesirable side effects [25]. An estimate by Alamgir [26] reveals that approximately 605 of the drugs that are available these days including paclitaxel, artemisinin, reserpine, and camptothecin were obtained from natural products either directly or indirectly.

Several studies exist where protein and phospholipid kinases have been targeted with the natural compounds. Most of the protein kinase inhibitors isolated from plants are polyphenols, mainly belonging to flavonoids [19–21, 27, 28]. However, there are representatives of nonflavonoids also that show inhibitory effects for human kinases, including resveratrol, caffeic acid, and gallic acid [29, 30]. Our previous studies demonstrated the binding and inhibition of CaMKIV by various natural compounds including hesperidin, *β*-carotene, and vanillin [31–33]. These compounds also showed the antiproliferative effect in breast (MCF-7), liver (HuH7 and HepG2), and neuroblastoma cancer cells (SH-SY5Y). These phytochemicals showed antiproliferative properties via inducing apoptotic pathways in cancerous cells.

Here, we studied a series of plant-derived natural compounds to assess their inhibitory effect towards CaMKIV [34]. The compounds showing best binding and inhibitory potential validated through experimental, docking, and molecular dynamics (MD) simulation studies. A detailed investigation of the binding mechanism provides atomistic insights into the mechanism of inhibition which may be further exploited for the design and development of clinical leads to address cancer and CaMKIV-associated diseases.

## 2. Materials and Methods

### 2.1. Materials

Isopropyl *β*-D-1-thiogalactopyranoside (IPTG), quercetin, ursolic acid, capsaicin, limonin, ellagic acid, simvastatin, DL-*α* tocopherol acetate, and dimethyl sulfoxide (DMSO) were procured from Sigma Aldrich (St. Louis, MO). Ni-NTA resin was bought from Thermo Scientific (USA). BIOMOL® Green reagent was bought from Enzo (New York, USA). All other reagents used were of analytical grade.

### 2.2. Molecular Docking Studies

The three-dimensional crystal structure of the CaMKIV enzyme was retrieved from the Protein Data Bank (https://www.rcsb.org/structure/2W4O). The structure of the enzyme was preprocessed, minimized, and refined using the Protein Preparation Wizard implemented in the Schrödinger suite [35–37]. This is associated with removing crystallographic waters, missing side chain or hydrogen atoms were added, and an accurate charge and protonation state was consigned to the enzyme structure consistent to pH 7.0 considering the applicable ionization states for the acidic as well as basic amino acid residues. The structure was subsequently subjected to energy minimization using the OPLS-2005 force-field [38] with an RMSD cut-off value of 0.30 Å to relieve the steric clashes among the residues due to the addition of hydrogen atoms.

The structure of quercetin (PubChem ID: 5280343) and EA (PubChem ID: 5281855) was retrieved from the PubChem database. These compounds were prepared using the LigPrep module implemented in the Schrödinger package [39]. This involved the addition of hydrogen atoms adjusting realistic bond lengths and angles, correct chiralities, ionization states, tautomers, stereo chemistries, and ring conformations. The OPLS-2005 force field was used to assign partial charges to the structure of the compounds. The compounds were subjected to energy minimization until average RMSD touched 0.001 Å. Epik [39] ionization tool was used to set the ionization state at the neutral pH.

Molecular docking was performed using the Glide [40] docking tool of Maestro 11.6 to identify the best binding energies suitable for the CaMKIV enzyme. Receptor grid was generated as center coordinates (*X* = 7.37, *Y* = 10.78, and *Z* = 15.03) using two cubical boxes having a common centroid to organize the calculations: a larger enclosing and a smaller binding box with dimensions of 15 × 15 × 15 Å. The grid box was centered on the centroid of the ligands in the complex, which was sufficiently large to explore a larger region of the enzyme structure. The ligands were docked by using the “Extra-Precision” mode (XP) protocol. The considered compounds were analyzed based on the docking scores and XP-G Scores.

### 2.3. MD Simulations

Molecular dynamics (MD) simulation was performed to see structural dynamics in the protein-ligand complex. The AMBER 18 package [41] was used to execute MD simulations on all the prepared systems using the GPU-accelerated simulation engine PMEMD. The atomic partial charges for the ligands were assigned using the ANTECHAMBER module employed in general amber force field (GAFF) [42, 43]. To entirely solvate each system in a TIP3P virtual box filled with water molecules within 8 Å box edge, the LEaP [44] component of AMBER 18 was utilized. The Cl^−^ and Na^+^ counter ions were added to approve the system neutralization before the production phase using the same LEaP component. A partial minimization of 1500 steps was achieved with 500 kcal/mol restraint potential gradient. The next full minimization of 1000 steps was further carried out by a conjugate gradient process by eliminating all the restraints applied before. Each system was then gradually being thermalized from 0 K to 300 K for 50 ps, assuring that each simulated system kept a stable volume and number of atoms. A potential harmonic restraint of 10 kcal/mol in combination with the collision frequency of 1 ps was applied to the solutes of all the systems. The equilibration of each system was performed by employing 500 ps equilibration step by ensuring 300 K constant temperature. NPT (isobaric-isothermal ensemble) was used to preserve a constant number of atoms and pressure within each system. The pressure was kept at 1 bar on each system using the Berendsen barostat. Ultimately, the production phase of 100 ns MD simulation was implemented on all the systems by integrating the SHAKE algorithm to restrict the hydrogen bonds [45].

### 2.4. Postdynamic Analyses

The coordinates of enzymes bound with the inhibitors were further saved after every 1 ps, and the trajectory curves were calculated using the CPPTRAJ [46] module integrated into the AMBER 18 package. The RMSD of C^*α*^ atoms, RMSF of each residue in the complex *R*_g_, SASA, intramolecular and intermolecular hydrogen bond formation, and thermodynamic calculations of all systems were calculated. We used Origin software and Gnuplot for MD trajectory analysis [47].

### 2.5. Binding Free Energy Calculations

The comparative binding free energies were analyzed using the Molecular Mechanics/Generalized Born Surface Area (MM/GBSA) binding free energy technique [48]. All solvent and counter ions were eliminated using the CPPTRAJ module. The binding free energies (Δ*G*_bind_) were estimated with the MM-GBSA method for each system as below:

(1) $$\begin{array}{c} \Delta G_{\text{bind}}=G_{\text{complex}}-G_{\text{protein}}-G_{\text{ligand}}. \end{array}$$

The free energy term, Δ*G*_bind_ is computed using the following equations:

(2) $$\begin{array}{c} \Delta G_{\text{bind}}=\Delta E_{\text{gas}}+\Delta G_{\text{solvation}}-T\Delta S, \end{array}$$

where

(3) $$\begin{array}{c} \Delta E_{\text{gas}}=E_{\mathrm{int}}+E_{\text{vdW}}+E_{\text{elec}}, \end{array}$$

(4) $$\begin{array}{c} E_{\mathrm{int}}=E_{\text{bond}}+E_{\text{angle}}+E_{\text{torsion}}, \end{array}$$

(5) $$\begin{array}{c} G_{\text{solvation},GB}=G_{\text{GB}}+G_{\text{nonpolar},\text{solvation}}, \end{array}$$

(6) $$\begin{array}{c} G_{\text{nonpolar}}=\gamma SASA+\beta . \end{array}$$

The gas-phase energy (Δ*E*_gas_) is the total of the internal (*E*_int_), van der Waals (*E*_vdW_), and Coulombic (Δ*E*_elec_) energies, (Equation (3)). The solvation free energy is the combination of polar (*G*_GB_) and nonpolar (Δ*G*_nonpolar_, Δ*G*_solvation_) contributions (Equation (5)). The polar solvation *G*_GB_ contribution was calculated using the Generalized Born (GB) solvation model with the dielectric constant 1 for solute and 80.0 for the solvent. However, the nonpolar free energy contribution was assessed using (Equation (6)), where the surface tension proportionality constant, *γ*, and the free energy of nonpolar solvation of a point solute, *β*, were set to 0.00542 kcal mol^−1^ Å^−2^ and 0 kcal mol^−1^, respectively. The SASA is calculated by the linear combination of the pairwise overlap (LCPO) model.

### 2.6. Dynamic of the Cross-Correlation Matrix

The dynamic cross-correlation matrix (DCCM) analysis was calculated to explore the variations and displacements in the C^*α*^ atoms. Components for *i* and *j* cross-correlation C^*α*^ atoms are presented underneath:

(7) $$\begin{array}{c} c_{ij}=\frac{<{∆r}_{i}.{∆r}_{j}>}{{\left(<{∆r}_{i}^{2}><{∆r}_{j}^{2}>\right)}^{1/2}}, \end{array}$$

where Δ*r*_*i*,*j*_ is the motion of *i*^th^ and *j*^th^ atom mean point, and angle braces indicate the interval throughout the complete curves. All correlated actions are denoted by *C*_*ij*_ = 1 whereas *C*_*ij*_ = −1 indicated highly anticorrelated motions over the course. The divergence of motion from 1 and -1 designate that *i* and *j* motions are correlated and anticorrelated correspondingly. The DCCM was analyzed using the CPPTRAJ module implemented in Amber 18 and all the matrices schemed in Origin software.

### 2.7. Expression and Purification of CaMKIV

The plasmid containing *CaMKIV* kinase domain cDNA (pET28a (+)) was transformed into an expression vector, BL21 (DE3) strain of *Escherichia coli*. The transformed *E*. *coli* cells were grown in Luria broth containing 50 *μ*g/ml kanamycin till the A_600_ reaches ~0.6-0.8. Subsequently, 0.25 mM IPTG was added to the culture broth followed by an incubation of 3-4 hrs at 37°C for the expression of recombinant protein. The detailed protocol for the purification of CAMKIV is described in our previous communications [18, 49]. Briefly, the cells were lysed by sonication, and inclusion bodies were prepared and subsequently solubilized using 1.0% of N-laurousyl sarcosine in CAPS buffer at pH 11.0. The protein was purified in a single step utilizing Ni-NTA affinity chromatography. The protein was eluted using 250 mM imidazole and dialyzed thoroughly against dialyzing buffer (20 mM phosphate buffer, pH 7.4, 100 mM NaCl) for 48 hours with 5-6 successive buffer changes yielding the refolded protein. A molar absorption coefficient of 47245 M^−1^ cm^−1^ at 280 nm was used to calculate the protein concentration using Jasco V-660 UV-visible spectrophotometer (Japan).

### 2.8. Fluorescence Spectroscopy

Fluorescence binding measurements were carried out on the Jasco fluorescence spectrophotometer (Jasco, Japan) equipped with a thermostated Peltier device that controls the temperature at 25 ± 0.1°C. A 5 mm quartz cuvette was used to perform all measurements. The compounds were dissolved in DMSO to prepare a 1 mM stock solution. CaMKIV (4.0 *μ*M in 20 mM phosphate buffer, pH 7.4) in the cuvette was titrated with increasing concentrations of the ligand from the stock solution. After each addition of ligand, the solution was properly mixed and the fluorescence emission spectrum was acquired from 300 to 400 nm wavelength range after exciting the protein at 280 nm. Both excitation and emission slits were set at 5 nm each. The corresponding blank was subtracted from each spectrum to obtain the final emission spectra. Subsequently, the Stern-Volmer equation was employed to analyze the data for the estimation of the number of binding sites (*n*) per molecule of CaMKIV and binding constant (*K*_*a*_) [50, 51].

### 2.9. Kinase Inhibition Assay

The ATPase activity of CaMKIV with ligands was evaluated by malachite green reagent (BIOMOL® Green reagent) -based microtitre plate assay. CaMKIV (2.0 *μ*M) was mixed with increasing concentrations of quercetin and EA and incubated for 30 min at 25°C. Subsequently, a freshly prepared ATP solution (200 *μ*M) was added followed by incubation for 30 min at 25°C. BIOMOL® Green reagent was then added gently to terminate the reaction followed by further incubation of 15 min for green color development. The microtitre plate was then read on ELISA reader at 620 nm. The standard phosphate curve was prepared as per the manufacturer's protocol to calculate the kinase activity in terms of inorganic phosphate released from ATP upon catalysis. Measurements in triplicates were performed as described [52].

## 3. Results and Discussion

### 3.1. Molecular Docking Analysis

**Figure 1:**
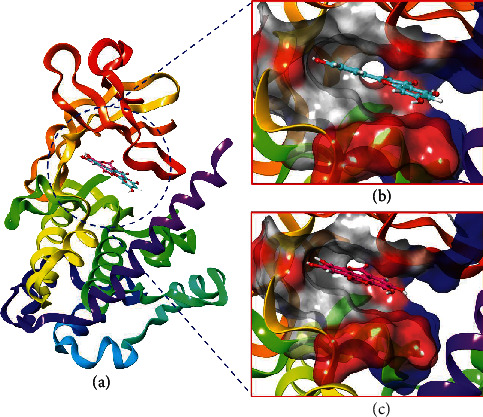
(a) Structural representation of CaMKIV in complex with quercetin (cyan color) and EA (pink color). (b) Close up surface view of the deep binding pocket of CaMKIV-accommodated quercetin. (c) Close up surface view of the deep active pocket accommodated with the EA (colored by residue type).

The natural compounds such as quercetin, EA, simvastatin, capsaicin, ursolic acid, DL-*α*-tocopherol acetate, and limonin were retrieved from the PubChem database to probe their potential interactions with the binding pocket of CaMKIV by molecular docking. We got an optimized orientation of ligands in the active site cavity of protein by minimizing the overall energies of the respective system. The calculated values of binding energies of all the compounds are shown in Table S1. Quercetin and EA showed the best binding energy and thus considered as promising inhibitors of CaMKIV (Figure 1). The quercetin-CaMKIV and EA-CaMKIV docked complexes showed significant binding energy values, -9.78 and -9.31 kcal/mol, respectively (Table S1.

**Figure 2:**
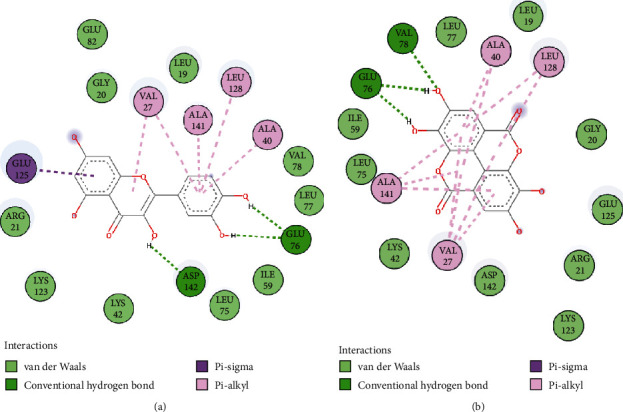
Docked poses of CaMKIV with the selected inhibitors. Molecular interaction of (a) quercetin and (b) EA with the active pocket of CaMKIV enzyme.

The interaction analysis of docked complexes suggests that both quercetin and EA form various stabilizing interactions including hydrophobic and H-bonded interactions with the residues of the active pocket of CaMKIV (Figure 2). Quercetin formed three hydrogen bonds with the residues of the active site pocket of CaMKIV. The catechol ring of quercetin forms two hydrogen bonds with polar side chains of Glu76, while the third hydrogen bond was formed by the hydroxyl (-OH) group of another ring with Asp142 (Figure 2(a)**)**. In addition, Glu125 is creating *π*-sigma interaction with the aromatic ring; whereas, a set of hydrophobic residues including, Val27, Ala40, Leu128, and Ala141 are forming a weak network of *π*-alkyl interactions with the catechol ring of quercetin.

The structure of EA is composed of a network of four aromatic rings that serve as a hydrophobic moiety, whereas the side chain lactone and hydroxyl groups act as an electron acceptor and H-bonding sites, respectively [53]. The polar sidechain of Glu76 forms two hydrogen bonds, while the backbone amide group of Val78 forms one hydrogen bond with the hydroxyl groups of EA (Figure 2(b)). A subset of hydrophobic residues having Val27, Ala40, Leu128, and Ala141 are forming a compact network of *π*-alkyl noncovalent interactions with the bulky aromatic rings of EA. Additionally, Asp142 is involved in stabilizing the complex via van der Waals interactions with EA.

Overall, the structural and interaction analyses revealed that both quercetin and EA bind strongly to the deep active site pocket of CaMKIV through hydrogen-bonded, *π*-sigma, and *π*-alkyl interactions that might be responsible for the modulation of enzyme activity.

### 3.2. Comparative Structural Dynamics

**Figure 3:**
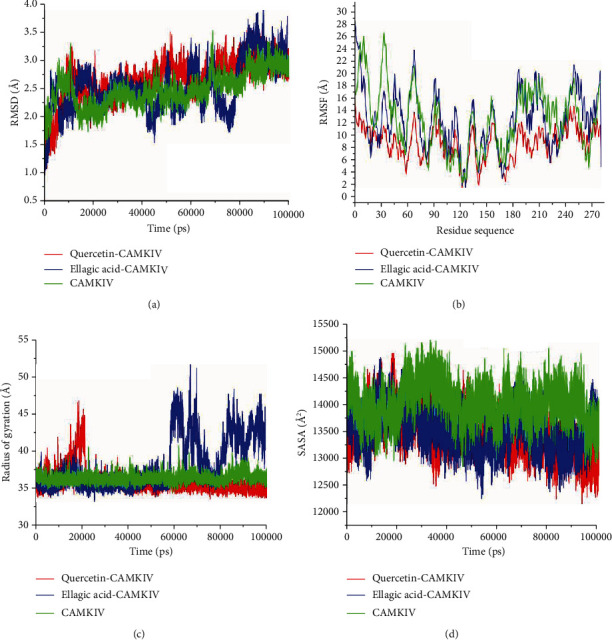
Structural dynamics of CaMKIV enzyme-ligand complexes. (a) RMSD, (b) RMSF, (c) *R*_g_ values, and (d) SASA values across C^*α*^ backbone in Å of quercetin- CaMKIV, EA-CaMKIV, and apo CaMKIV; in Å across C^*α*^ backbone of all the three conditions calculated during the 100 ns of MD trajectories.

The conformational changes in the enzyme structure are precisely coupled with their biological functions [54]. Any alteration or interruption in enzymes' structural integrity could have a considerable impact on its function [55]. The binding of small molecule inhibitors influences the mode of action of enzymes that are implicated in disease pathways; hence, it is required to evaluate the structural dynamics and structural changes associated with the inhibitory activity of these compounds [56]. The calculation of a time variable concerning the root means square deviation (RMSD) across C^*α*^ atoms from generated trajectories were executed to determine the consistency and efficiency of the simulated CaMKIV in complex with quercetin and EA along with the CaMKIV alone [57]. The alterations in the RMSD values were shown in the plot throughout the simulation period, possible conformational changes within the structure of an enzyme upon ligand binding. As Figure 3(a) suggests, all the systems were stabilized and achieved a convergence after 40 ns of the production phase of the simulation. Quercetin-CMKIV complex exhibited the lowest average RMSD of 2.47 Å, whereas EA-CaMKIV and apo CaMKIV showed an average RMSD of 2.49 Å and 2.51 Å, respectively. The RMSD plots indicate that both quercetin and EA exhibit almost the lowest change in the C^*α*^ backbone atoms, suggesting binding of these compounds induces stability to the CaMKIV structure. The overall inhibitory activity of CaMKIV by quercetin and EA was supported by structural stabilization and subsequent inhibition in the enzyme activity. The stability of quercetin and EA were further assessed throughout the simulation time as their related activities may correspond to the active pocket residues.

The flexible regions of a protein provide flexibility which is sometimes very important for the biological activity especially in the case of the enzyme [58]. Hence, ligand-enzyme binding may be observed concerning the alteration in flexibility expressed in terms of root mean square fluctuation (RMSF). To ascertain the flexibility and rigidity of all the residues in CaMKIV upon quercetin and EA binding, RMSF values for C^*α*^ atoms were estimated through the MD simulations produced trajectories over 100 ns of the period. As illustrated in Figure 3(b), the quercetin-CaMKIV complex revealed a minimum fluctuation in the residues with an average value of 8.45 Å. Standard RMSF values of 11.14 Å and 12.29 Å were observed in EA-CaMKIV complex and apo CaMKIV, respectively. These observations revealed that quercetin exhibited the lowest fluctuation which could be indicative of a better binding in comparison to the EA. This considerable decrease may be associated with structural deactivation that was assured as a result of the prominent binding of compounds in the active pocket of CaMKIV. A reduced fluctuation of all the residues might have favored CaMKIV inhibition.

To further complement our analysis, we have estimated *R*_g_ value, related to the intact conformational changes in the enzyme structure upon ligand binding. It also uncovers the compactness, stability, and folding behavior of enzyme structure [59]. We assessed the compactness of quercetin, EA, and apo CaMKIV complexes by evaluating their *R*_g_ values. The average *R*_g_ values for quercetin-CaMKIV, EA-CaMKIVcomplexes, and apo CaMKIV were calculated as 37.12 Å, 41.39 Å, and 36.12 Å, respectively.  Figure 3(C) showed a slight alteration in the compactness in the presence of two compounds. Quercetin showed a higher fluctuation before 20 ns, but afterward, it was stable. EA also exhibited similar behavior as it showed stability until 60 ns and after that higher fluctuation was noted. The lowest *R*_g_ value was exhibited by the apo CaMKIV enzyme, suggesting no significant change upon ligand binding to the CaMKIV enzyme.

We further analyzed changes in the SASA values to define the hydrophobic and hydrophilic function of residues and forces exposed to the solvent in the simulation period [60, 61]. The rapid and constant values of SASA is advantageous in the energetic assessment of biomolecules. The connection among native hydrophobic contacts inside the enzyme structure is an important intermolecular interaction which is associated with the enzyme inhibition. Hydrophobic interaction formed between the nonpolar residues endorses constancy of the enzyme structure within the solution by preserving the nonpolar residues inside the hydrophobic core away from an aqueous solution [62].  Figure 3(D) illustrates overall changes in the SASA values for all the complexes analyzed during 100 ns of MD simulation time. The average SASA value for the quercetin-CaMKIV complex was 13512 Å^2^ exposed to the solvent system. The total value of SASA of 13683 Å^2^ and 14079 Å^2^ was calculated for EA-CaMKIV complex and apo CaMKIV, respectively. Such a difference in the values of SASA is related to the extent of binding and their subsequent impact on the enzyme inhibition. The assessment of SASA in the quercetin-bound complex revealed a better exposure to the solvent and consequently favored the increased inhibitory potential of quercetin over other complexes.

### 3.3. Analysis of Intermolecular and Intramolecular Hydrogen Bonds

**Figure 4:**
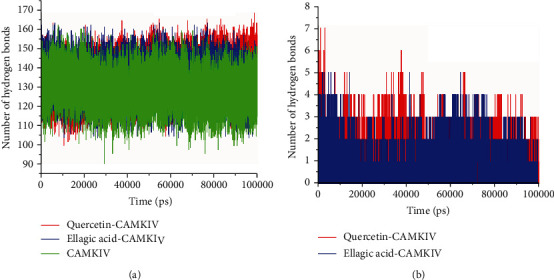
Hydrogen bond analysis. (a) Intramolecular hydrogen bonds in quercetin, EA-CaMKIV complexes, and apo CaMKIV enzyme. (b) Intermolecular hydrogen bonds in quercetin, and EA-bound CaMKIV complexes calculated after 100 ns MD simulation.

We have calculated the intramolecular and intermolecular hydrogen bonding for CaMKIV alone and in complex with quercetin and EA to confirm the overall stability and strength of interactions [40].  Figure 4 provides a detailed information of the hydrogen bonding pattern of protein-ligand interactions. In the quercetin-CaMKIV complex, an average number of intramolecular hydrogen bond formation was noted as 137; however, in the case of EA-CaMKIV and apo CaMKIV, it was 135 and 130, respectively, during the 100 ns simulation time. On the other hand, the intermolecular hydrogen bond formation in the active pocket of CaMKIV enzyme observed to be 3-4 bonds with higher fluctuations and 2-3 bonds with the least fluctuations in quercetin and EA complexes.

### 3.4. Secondary Structure Analysis

**Figure 5:**
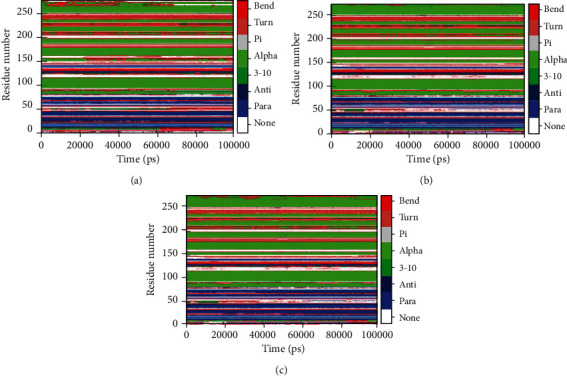
Secondary structure analysis. (a) Apo CaMKIV enzyme. (b) Quercetin-bound CaMKIV enzyme. (c) EA-bound CaMKI enzyme.

To analyze the alterations incurred due to ligand binding to the CaMKIV enzyme, the dynamics of secondary structure contents were measured. The structural elements like *α*-helix, *β*-sheet, and turn in CaMKIV were classified into individual amino acid residues for every time step; thus, an average of amino acid residues involved in the formation of secondary structure was calculated.  Figure 5(a) shows that in apo CaMKIV marginally a higher fluctuation in the turn in comparison to the other structural components. A slight increase was observed in the *α*-helix and bend in quercetin and EA-bound CaMKIV complexes (Figures 5(b) and 5(c)). These higher components are suggestive of a stabilized binding of the ligands to the enzyme. Although secondary structure components in CaMKIV do not contribute to any major changes showing enhanced flexibility, compactness, and stability of both the compounds.

### 3.5. Dynamic Cross-Correlation Matrix Analysis

**Figure 6:**
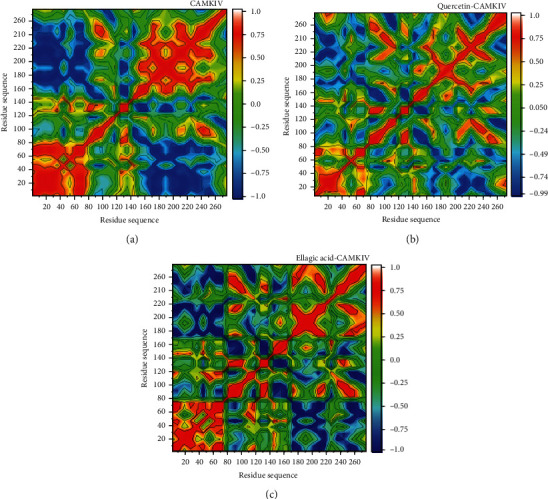
Dynamic cross-correlation matrix analyses. (a) Apo CaMKIV enzyme. (b) Quercetin-bound CaMKIV complex. (c) EA-bound CaMKIV enzyme inhibition. Numbers closer to 1 indicate high correlation, while those closer to -1 indicate anticorrelation between pairs of residues.

Distinct enzyme dynamics among apo CaMKIV, quercetin-CaMKIV, and EA-CaMKIV complexes were measure by plotting dynamic cross-correlation matrix for the correlated and anticorrelated motions of all residues. CaMKIV was separated into different groups with positive and negative movements of amino acid residues. The apo CaMKIV was showing both positive and negative correlation between the residual motions (Figure 6(a)). As shown in Figures 6(b) and 6(c), significant differences were observed between quercetin and EA-CaMKIV complexes. There was a less positive correlation in the quercetin-bound complex as compared to the EA complex. The apo CaMKIV and EA-CaMKIV complex appeared to be similar, indicative that inhibition through EA induced noteworthy changes in CaMKIV dynamics. Few residues (1-40 and 260-302) in quercetin-CaMKIV complex showed a highly positive correlation of movements; therefore, they might be contributing to the overall dynamics of the CaMKIV enzyme.

### 3.6. Thermodynamics Free Energy Landscape

The thermodynamic energy contribution of a ligand to the overall binding free energy of the complex is directly related to the structural stability of the ligand in the active pocket of the enzyme. The molecular interactions of residues in the active pocket engage significantly in the stability, binding affinity, and selectivity of the ligand. Thus, it was essential to assess the binding affinity of quercetin and EA towards the CaMKIV enzyme using the MM/GBSA approach to determine the effect of these compounds. The obtained results are displayed in Table 1.

The free binding energy (Δ*G*_bind_) of quercetin-bound CaMKIV complex was noted to be the highest energy with an average value of -29.15 kcal/mol relative to the EA-CaMKIV complex with -23.75 kcal/mol. These total binding energies of the complexes were indicative of the effective binding of quercetin to its target enzyme. We further assessed other constituents of the free binding energy coupled with enzyme-inhibitor binding (Table 1). It was observed that intermolecular van der Waals and gas-phase energies were more favorable in the EA-CaMKIV complex with the average values of -33.69 and -60.20 kcal/mol, whereas, these energies are slightly less in quercetin bound CaMKIV (-25.90 and -55.32 kcal/mol). There was a significant difference of -5.4 kcal/mol between the Δ*G*_bind_ energies of quercetin and EA complexes. The Δ*G*_solvation_ contributed to unfavorable binding of quercetin as it was the least energy with a value of 26.18 kcal/mol between the two complexes. However, the systematic motions of quercetin and EA from the solvent phase to the active pocket of CaMKIV-stimulated van der Waals and electrostatic interactions with the active site residues, but these interactions were not adequate to support completely in the binding of the CaMKIV enzyme as Δ*G*_bind_ contributes to the improved binding of these compounds. This analysis suggests that both quercetin and EA possess a significant binding affinity to the CaMKIV.

### 3.7. Per Residue Energy Decomposition Analysis

**Figure 7:**
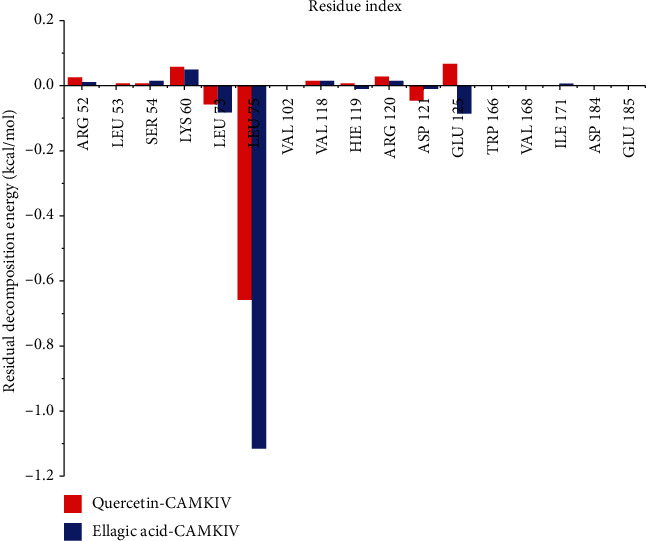
The per residue energy decomposition analysis graphs of quercetin and ellagic acid CaMKIV complexes, plotted after 100 ns MD simulations.

The binding free energy decomposition gives a deeper insight into the annotation of enzyme-ligand complexes generated through the trajectories by MD simulations. Herein, we have disintegrated the overall binding energies of complexes into per residual contribution by each amino acid residue present in the active pocket of the CaMKIV enzyme to get detailed insights into key residues involved in ligand binding. The interactions among active site pocket electronegative and electropositive residues improve ligand binding and its stabilization at the target site. This forms an improved intermolecular binding that upsurges the binding affinity of the ligand in the active pocket. Leu76 contributed with the lowest Δ*G*_bind_ with -1.76 kcal/mol in EA binding, whereas this residue has contributed with slightly less Δ*G*_bind_ of -0.67 kcal/mol in quercetin-bound complex (Figure 7). The Δ*G*_bind_ of another contributing residue Leu73 was also lower in the case of EA-CaMKIV with -0.45 kcal/mol; however, it is marginally less in the quercetin-CaMKIV complex (-0.35 kcal/mol). Arg52, Lys60, Val118, and Arg120 contributed to the binding of quercetin and EA with positive binding energies. Glu125 amino acid residue contributed to the negative energy of -0.05 kcal/mol in EA, whereas this residue was showing positive energy of 0.03 kcal/mol in quercetin-bound CaMKIV complex. Our analysis reveals residues contributing to the overall binding energies of both the complexes.

### 3.8. Fluorescence Binding Studies

Fluorescence quenching has long been utilized to measure the binding affinity of the protein with the ligand [63, 64]. An array of molecular interactions such as intersystem crossing to the triplet state, formation of an excited charge-transfer complex, complex formation between the quencher and fluorophore at ground state, and molecular rearrangements can be attributed to fluorescence quenching [65].

**Figure 8:**
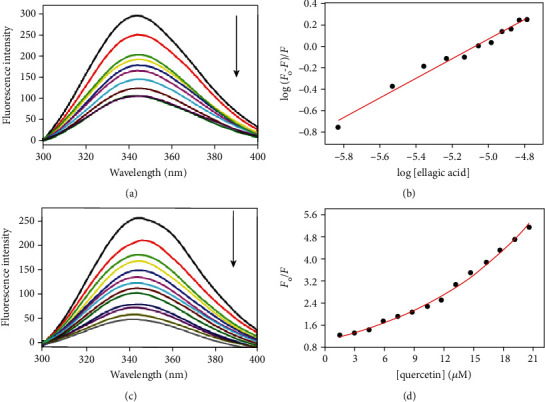
Fluorescence binding study of CaMKIV with quercetin and EA. (a) Fluorescence spectra of CaMKIV (4 *μ*M) with increasing concentrations of EA (from top to bottom) at pH 7.4. (b) Stern-Volmer plot obtained by plotting fluorescence intensity at *λ*_max_ as a function of EA and fitting to Equation (1). (c) Fluorescence spectra of CaMKIV (4 *μ*M) with increasing concentrations of EA (from top to bottom) at pH 7.4. The excitation was kept constant at 280 nm and the emission spectra was acquired from 300 to 400 nm. (d) Stern-Volmer plot obtained by plotting fluorescence intensity at *λ*_max_ as a function of quercetin fitting to Equation (2).

To complement the *in silico* findings, we carried out fluorescence measurements to determine the binding affinity of CaMKIV with the selected natural compounds (Figure S1). CaMKIV (4.0 *μ*M) was titrated with an increasing concentrations of natural compounds from 1.0 mM stock solution. Thereafter, fluorescence emission spectra were collected in the wavelength range of 300 to 400 nm after exciting the protein at 280 nm. The titration and data acquisition was followed until the saturation point was achieved.  Figure 8 and Figure S1 shows the emission spectra of CaMKIV in the presence of an increasing concentration of different ligands. The compounds showing good binding interaction with CaMKIV are quercetin and EA; a continual decline in fluorescence intensity was noticed with each successive addition of ligand (Figures 8(a) and 8(c)). In contrast, the other compounds showed less or no significant quenching effect indicating the absence of any strong binding interaction with the protein (Figure S1). For EA and quercetin, the fluorescence intensity at *λ*_max_ (*F*_0_/*F*) was plotted against a ligand concentration and fitted to the Stern-Volmer equation to estimate binding constants (*K*sv) (Figures 8(b) and 8(d)).

The following equations were used to analyze data depending on the resulting curves and quenching mechanism [66]:

(8) $$\begin{array}{c} \log\left(F_{0}-F/F\right)=\log\;{\text{K}}_{a}+n\log\left[Q\right], \end{array}$$

(9) $$\begin{array}{c} F_{0}/F=a^{*}\exp\left({K_{\text{sv}}}^{*}Q\right), \end{array}$$

where *F*_0_ and *F* are the fluorescence intensities in the absence and presence of ligands, respectively; *K*_*a*_ and *K*_sv_ depict binding constants; *n* is the number of the binding site(s); and *a* is the amplitude.

For EA, a linear model as depicted in Equation (8) was used. In contrast, a nonlinear model as described by Equation (9) was applied for quercetin to fit the Stern-Volmer plot with the upward curvature having exponential dependence. This deviation from linearity (i.e., upward curvature) in the case of quercetin can be attributed to the occurrence of both static and dynamic quenching and the differential binding of the ligand to the protein molecule. The values of binding constants obtained were 4.28 × 10^4^ M^−1^ (*K*_sv_) and 7.82 × 10^2^ M^−1^ (*K*_sv_) for EA and quercetin, respectively (Table 2). The other natural compounds do not quench the intrinsic fluorescence of CAMKIV appreciably (Figure S1), and hence, they were not evaluated further in the search to identify the potent inhibitors of CaMKIV.

### 3.9. Enzyme Inhibition Assay

**Figure 9:**
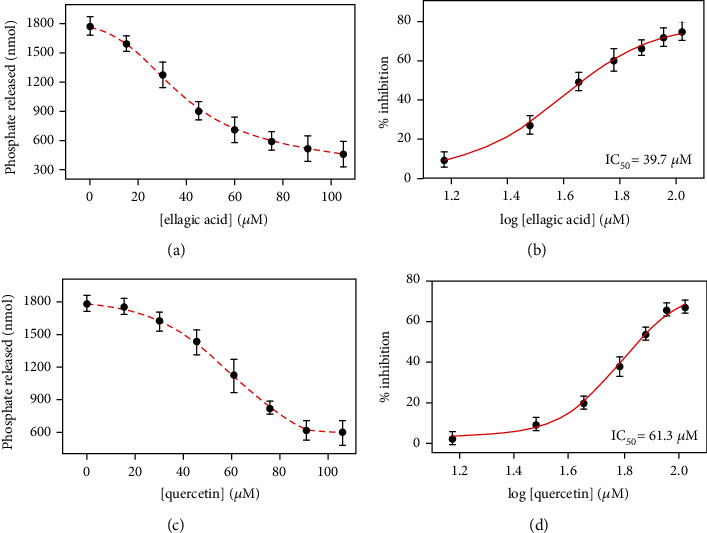
ATPase inhibition assay of CaMKIV with EA and quercetin. (a) Dose-response curve showing the effect of increasing concentrations of EA (0 to 100 *μ*M) on the kinase activity of CaMKIV. The standard phosphate curve was used to calculate the amount of inorganic phosphate released from ATP in a reaction catalyzed by CaMKIV. (b) Plot depicting the amount of % inhibition in ATPase activity as a function of log [EA]. The data were fit to estimate the value of IC50 for EA using GraphPad Prism 5.0. (c) The dose-response curve showing the effect of increasing concentrations of quercetin (0 to 100 *μ*M) on the kinase activity of CaMKIV. (d) Plot depicting the amount of % inhibition in ATPase activity as a function of log [quercetin]. Results shown here are depicted as means ± SD of three independent experiments.

CaMKIV possess an inherent ATPase activity [67, 68]. Thus, we have employed BIOMOL reagent-based kinase assay wherein the inorganic phosphate released by the hydrolysis of ATP in a reaction catalyzed by CaMKIV forms a green-colored complex with malachite green. The complex absorbs at 620 nm that gives an estimate about the catalytic activity of CaMKIV. The inhibition assay was performed in the presence of different concentrations of EA and quercetin to evaluate their capacity to inhibit the enzyme activity of CaMKIV. Figures 9(a) and 9(c) shows dose-responses curve obtained by plotting the amount of phosphate released by the ATPase activity of CaMKIV as a function of ligand concentrations. A continuous decrease in the enzyme-catalyzed ATP hydrolysis was observed in a dose-dependent manner for both EA and quercetin. Around 70% decrease in the ATPase activity of CaMKIV was noticed in the presence of EA and quercetin. The raw data were converted to % inhibition values using the formula, 100–(*A*/*A*_0_ × 100) where *A*_0_ and *A* represent the enzyme activity of CaMKIV in the absence and presence of the compound. The percent inhibition in kinase activity was plotted against log [compound], and data were fitted to estimate the value of IC_50_ (50% of ATPase inhibition) for both the compounds using GraphPad Prism 5.0 (Figures 9(b) and 9(d)). The IC_50_ values obtained for EA and quercetin were 39.7 and 61.3 *μ*M, respectively. It is quite evident from IC_50_ values, that both EA and quercetin are potent in inhibiting the catalytic activity of CaMKIV in the micromolar range. All together, the results from fluorescence binding and enzyme activity show that EA and quercetin act as potential inhibitors of CaMKIV with appreciable binding affinity.

## 4. Conclusions

We conclude that both EA and quercetin bind to the active site pocket of CaMKIV and thus serve as potent inhibitors. They form H-bonded interactions with active site residues, thereby significantly reduces the catalytic activity of CaMKIV. Thus, targeting CaMKIV by these phytochemicals can be a potential curative strategy for the treatment of cancer and other human diseases linked to the abnormal expression of CaMKIV. The usage of EA and quercetin as drug-like molecules can be further explored by making suitable modifications on the basic scaffold to improve their bioavailability and therapeutic potential. Overall, we believe that this study widens the scope of the utilization of natural compounds in the development of therapeutics for various human diseases including cancer.

## Figures and Tables

**Table 1:** MM/GBSA-based binding energy profile of CaMKIV in complex with quercetin and EA.

Complex	Δ*E*_vdW_	Δ*E*_elec_	Δ*G*_gas_	Δ*G*_polar_	Δ*G*_nonpolar_	Δ*G*_solvation_	Δ*G*_bind_
Quercetin-CaMKIV	-33.69	-26.51	-60.20	41.03	-4.58	36.45	-23.75
EA-CaMKIV	-25.90	-29.42	-55.32	30.43	-4.26	26.18	-29.15

**Table 2:** Binding constants and inhibitory concentrations of EA and quercetin to CAMKIV.

Compound	^∗^Binding constant (*K*_sv_, M^−1^)	^¥^IC_50_ (*μ*M)
EA	4.28 ± 0.28 × 10^4^	39.7 ± 8.6
Quercetin	7.82 ± 0.54 × 10^2^	61.3 ± 5.4

^∗^Binding constant derived from fluorescence binding measurements. ^¥^IC_50_ value determined from enzyme inhibition assay.

## Data Availability

The data used to support the findings of this study are included within the supplementary information files.

## Supplementary Materials

Supplementary Materials Figure S1: fluorescence binding study of CaMKIV with natural compounds. Fluorescence emission spectra of CaMKIV (4 *μ*M) with increasing concentrations of compounds at pH 7.4. (a) Capsaicin. (b) Limonin. (c) Simvastatin. (d) DL-*α* tocopherol acetate. (e) Ursolic acid. Table S1: docking *g* score of selected and screened natural compounds with their binding site residues.
