# Supplementary material for: Identification of Potential Inhibitors of Calcium/Calmodulin-Dependent Protein Kinase IV from Bioactive Phytoconstituents
Source: Oxid Med Cell Longev. 2020 Jul 16;2020:2094635. doi: 10.1155/2020/2094635 (PMC7382742; doi:10.1155/2020/2094635)
Supplement: Supplementary Materials — Figure S1: fluorescence binding study of CaMKIV with natural compounds. Fluorescence emission spectra of CaMKIV (4 μM) with increasing concentrations of compounds at pH 7.4. (a) Capsaicin. (b) Limonin. (c) Simvastatin. (d) DL-α tocopherol acetate. (e) Ursolic acid. Table S1: docking g score of selected and screened natural compounds with their binding site residues. [file 2094635.f1.doc]

**Supplementary information**

Identification of potential inhibitors of Calcium/calmodulin dependent protein kinase IV from bioactive phytoconstituents

Preeti Gupta ^1^, Shama Khan^2^+, Zeynab Fakhar^3^, Afzal Hussain^4^, Md. Tabish Rehman^4^, Mohamed F. Alajmi^4^, Asimul Islam^1^, Faizan Ahmad^1^ and Md. Imtaiyaz Hassan^1^,*

*^1^Centre for Interdisciplinary Research in Basic Sciences, Jamia Millia Islamia, Jamia Nagar, New Delhi 110025, India;* [*fun.preets@gmail.com*](mailto:fun.preets@gmail.com) *(P.G.);* [*aislam@jmi.ac.in*](mailto:aislam@jmi.ac.in) *(A.I.);* [*fahmad@jmi.ac.in*](mailto:fahmad@jmi.ac.in) *(F.A.)*

*^2^Department of Clinical Microbiology and Infectious Diseases, School of Pathology, University of Witwatersrand, Johannesburg 2193, South Africa;* [*shama.khan@wits.ac.za*](mailto:shama.khan@wits.ac.za) *(S.K.)*

*^3^Molecular Sciences Institute, School of Chemistry, University of the Witwatersrand, WITS 2050, Johannesburg, South Africa;* [*zb.fakhar@gmail.com*](mailto:zb.fakhar@gmail.com) *(Z.F.)*

*^4^Department of Pharmacognosy, College of Pharmacy, King Saud University, Riyadh 11451 KSA.* [*afzal.hussain.amu@gmail.com*](mailto:afzal.hussain.amu@gmail.com) *(A.H.),* [*mrehman@ksu.edu.sa*](mailto:mrehman@ksu.edu.sa) *(M.T.R.),* [*malajmii@ksu.edu.sa*](mailto:malajmii@ksu.edu.sa) *(M.F.A.)*


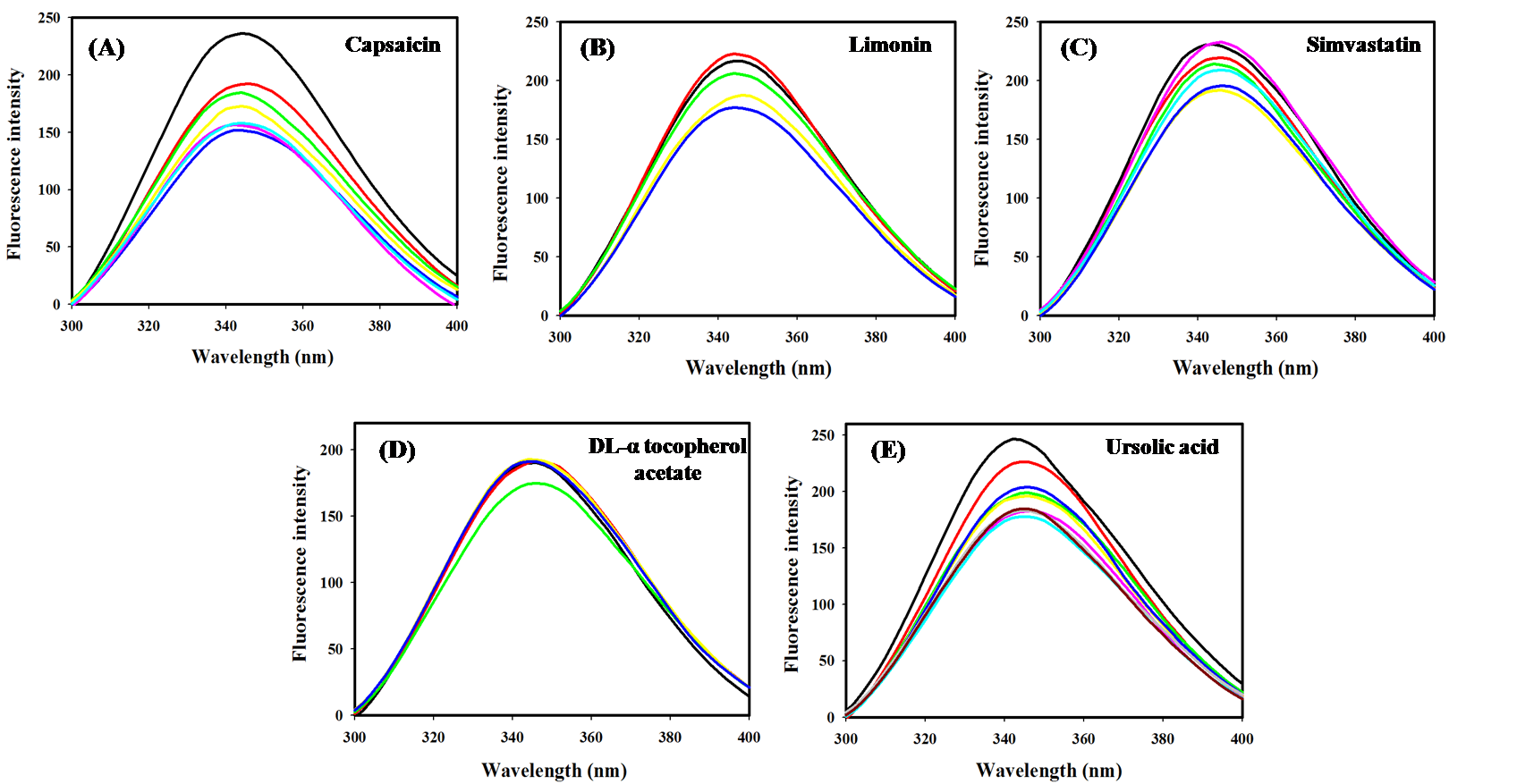


**Figure S1:** **Fluorescence binding study of CaMKIV with natural compounds.** Fluorescence emission spectra of CaMKIV (4 µM) with increasing concentrations of compounds at pH 7.4. **A.** Capsaicin **B.** Limonin **C.** Simvastatin **D.** DL-α tocopherol acetate **E.** Ursolic acid

**Table S1:** Docking gscore of selected and screened natural compounds with their binding site residues.

| **Compounds** | **Docking score**  **(kcal/mol)** | **Interacting Binding Site Residues** |
| --- | --- | --- |
| Quercetin | -9.78 | Val60, Asp185, Ala184, Lys75, Ile102, Ala73, Leu118, Glu119, Leu120, Val121, Lys166, Glu168, Leu171, Arg54, Gly53, Leu52, Glu125 |
| Ellagic acid | -9.31 | Val60, Lys75, Asp185, Ala184, Ala73, Leu118, Ile102, Glu119, Leu120, Val121, Leu171, Glu168, Lys166, Leu52, Gly53, Arg54 |
| Simvastatin | -5.59 | Asp164, Lys166, Glu168, Asn169, Leu 171, Ala184, Asp185, Leu52, Gly53, Arg54, Gly55, Thr57, Ser58, Ile59, Val60, Lys75, Ala73, Ile102, Leu118, Leu120, Val121, Gly124, Glu125 |
| Capsaicin | -5.55 | Val60, Ser58, Thr57, Gly55, Arg54, Gly53, Leu52, Ala184, Asp185, Lys166, Glu168, Asn169, Leu171, Gly124, Gly123, Thr122, Val121, Leu120, Glu119, Leu118, Ile102, Lys75, Ala73 |
| Ursolic acid | -3.01 | Glu168, Leu171, Arg54, Gly53, Leu52, Val60, Arg62, Glu125, Gly124, Gly123, Thr122, Val121, Leu120, Pro175 |
| DL-α tacopherol acetate | -3.009 | Ala328, Lys331, Glu125, Gly124, Thr122, Val121, Leu120, Leu118, Ile102, Asp185, Ala184, Lys75, Ala73, Lys166, Glu168, Asn169, Leu171, Arg621, Val60, Ser58, Ala56, Gly55, Arg54, Gly53, Leu52, Gly203, Thr204 |
| Limonin | -2.63 | Glu168, Leu171, Leu120, Val121, Thr122, Gly123, Gly124, Glu125, Val60, Arg62, Leu52, Glu51, Ser50 |
